# Rheumatism

**Published:** 1897-02

**Authors:** E. B. Jackson

**Affiliations:** Houston


For the Texas Medical Journal.

### RHEUMATISM.

BY E. B. JACKSON, M. D., HOUSTON.

RHEUMATISM is a constitutional disease, cropping out by an inflammatory process in the joints, and in the fibrous texture of other parts, and showing the capability of producing many and varied complications. It is accompanied by much pain, fever, and a clamminess of the skin. It shows a tendency to spread from joint to joint, in a manner that is not very well understood.

There is much dissention among writers as to the nature of the disease. Many theories have been deduced from experimental evidence, some of which have been scientifically and ingeniously supported by practitioners of wide experience and great renown. None of them, in point of fact, have been sufficiently verified by clinical observation and experience to gain universal favor.

Lactic acid in excess in the system, as the result of dyscrasia of the nutrition process, is one theory. Chill of the joint, received locally, through the blood or nervous system, by which its nutrition is disturbed, may be, and often is, followed by textural inflammation. Again, it is said that the heat regulating mechanism of the body, may be so extensively affected by a chill that the muscular system may abnormally produce and abundantly store up heat, conveying, consequently, to the brain the impressions of pain by the articular nerves.

Still again, it has been set forth that rheumatism is the result of some germ or some miasmatic poison in the blood. It is not in the province of this paper to discuss the relative merits of these theories.

It is generally agreed that a tendency is conveyed to posterity by those afflicted with rheumatism, and that their offspring fall easy victims to the exciting causes of the disease. Hereditary influences are thus easily and authentically traced in one-fourth of all cases. This disease is not strictly confined to young subjects, but by far the greater number of cases are met with in individuals between the ages of fifteen and twenty-five. It may occur in early childhood; it may occur in old age, though rarely. It is more commonly met with in the poorer classes. It is specially liable to affect those who are forced to stem hard weather, those whose lives are filled with habitual anxiety, overwork and exposure, those who have been long subjected to depressing influences. It is a well known fact that the disease most frequently occurs in a damp, cold climate, and among the class who are not warmly clothed, well fed. and rested. The disease is, however, met with in New Mexico and everywhere. The onset of rheumatism is usually preceded by a cold drenching with rain, by exposure to cold draughts of damp air, or by the fatigue of excessive exercise rapidly followed by chill.

All those persons who have once sustained the disease, are more prone to a second attack when subjected to exciting causes, and the length of time will shorten between all successive attacks; rigors and chilly sensations, usher in the disease. The rigors are almost instantly followed by a considerable rise in the body temperature; there is intense pain and much stiffness in the joint or joints affected. Often the fever runs high; there is restlessness, helplessness, prostration, fear of being touched, dread of the least jolt, complete loss of energy. There is a sour sweat exuding from the body; the pulse is full, soft and rapid; temperature probably 105; the face flushed; the expression one which is at once indicative of pain. The bowels are constipated; there is no appetite; the tongue has a yellow fur on it; there is thirst, and the patient is generally miserable. The urine is full of urates, intensely acid, and scanty. The pain may be at first confined to but one joint. Soon, however, others will probably become affected. In extending to new fields the disease usually prefers to attack the mate-joint of the opposite side of the body. After the inflammation has extended to other joints, the lesion may, in great measure, subside in the one first affected, but may recur at any time prior to general subsidence of the disease. In the severer forms of rheumatism nearly all of the joints are traversed by the inflammatory process, in which event the condition of the patient is rendered both miserable and precarious. There is no means of estimating the probable duration of an attack of acute rheumatism. In some cases the symptoms will subside short of ten days, whereas in many cases they may persist until even months have been recounted, and the case merged into one of chronic rheumatism in which all of the acute symptoms have disappeared with the exception of pain and stiffness, which are now slight, and are only disagreeable when the afflicted member is brought into motion. The patient is now emaciated, pale and weak, and subject to recurring attacks upon the slightest indiscretions. If the case should have pursued a mild course, ending in a few days from its inception, the patient may be left completely cured, though the liability to a recurrence of the disease doubtless remains with him forever. If in the course of the disease, hyperpyrexia should occur, as at any time may happen, the case at once becomes dangerous and requires prompt attention to prevent speedy death. The temperature may very suddenly go to 108 or 110. It is when life is thus endangered that heroic measures should be instantly applied. Chorea is occasionally seen in connection with this disease. There is sometimes delirium and excitement.

Along with these head symptoms there may be pulmonary and bronchial disturbances in the form of pleurisy, pneumonia or bronchitis. Of all the complications, however, the heart plays the most important role, eudo carditis and pericarditis being extremely liable to occur at any moment, therefore the ever present anxiety in reference to acute cases in quite young subjects, when there is always much danger of the foundation of serious organic heart disease being laid.

Sub-acute rheumatism, as its name implies, is a mild type of the disease, and while it may persist for a greater length of time, and seem to be less amenable to treatment, the dangers of heart complications are probably much less than in the acute variety. It is agreed that chronic rheumatism not infrequently follows in the wake of acute cases, but it is oftener developed independently. It is more often hereditary, and comes on in later life, and, as in the acute form, it is exited by cold, exposure, overwork, or any form of privation or unhealthy habits tending to depress and debilitate the vital forces. Thus it may afflict lead-workers, drunkards, or syphilitics. While it is a constitutional disease, the systemic disturbances are much less than in the acute variety, and are not so likely to be followed by the various serious complications. In this form of the disease, however, the joint injury is more permanent, in some instances reaching virtually complete ankylosis, and in the event the sheaths of the muscles and nerves partake of the morbid process, there will be great disability with intense and long suffering.

This form of the disease is not nearly so liable to change from joint to joint, preferring to fix itself usually in some of the joints which are the most useful, and likewise the most exposed; in the hands and feet.

Many of us know from experience how much easier we can walk, and how the pain and stiffness partly passes away after a little limbering up with exercises; also how easy it is to foretell the probable state of the weather on the forthcoming day. Humid atmosphere, in many cases, will ache the joints, a dry, crisp air the contrary, so that nearly every rural vicinity has its weather prophet, whose success furnishes him with the respect of his neighbors. It is a fact, and not a myth of legendry lore, but it is well to remember that all signs fail in dry weather.

Rheumatism of this character has a very telling effect upon the constitution of its subject, rendering his general health poor, his body puny, and likewise more prone to the contraction of other diseases, though it is not to be considered as immediately dangerous to life. It often produces unsightly and lamentably enormous deformities, rendering its victim almost helpless—sometimes, indeed, quite helpless. Surgical interference may relieve some of these deformities, and it is quite justifiable, inasmuch as the stiffness is permanent in all cases where the integrity of the joint is hopelessly impaired.

Suppose a patient comes, complaining of a toe, which, for one year has been drawn to its utmost limit under the foot; there is much pain and inconvenience as may at once very easily be seen. A case of this character came to my notice lately, and the member was immediately amputated. Another case which will be mentioned as occurring in the author's family, in which the right index finger was removed for an aunt, who was deprived of the use of the entire limb by reason of the pain and deformity of the offending digit. Much good in this way may be accomplished, not only in that the sufferers are given instant surcease of pain, but are likewise rendered capable of doing efficient work in their several callings.

In reference to the treatment of acute rheumatism, the general directions are, first, a warm room and bed, and the patient should quietly reside between a comfortable pair of light blankets. The affected joints should be evenly and lightly bandaged after being snugly wrapped in cotton-wool batting. This dressing may be removed in order that the parts may be gently rubbed with a warm sedative of any character that may prove grateful and effective. Hot olive oil containing a strong per centage of laudanum may be of service.

Without attempting to enter into a general discussion of the various drugs which are of supposed utility, the treatment first proposed by Dr. Maclagan will be mentioned, viz: Salicin, salicylic acid, and salicylate of soda. These remedies are known to relieve pain, reduce fever, and in many instances shorten the attack before the alarming complications have had time to supervene. The administration of the salicylates is recommended in simple doses every two hours for the first day and night, at the end of which time the general salutary impression is usually witnessed, whereupon a reduction of the dosage should be practiced. If there is marked subsidence of pyrexia and pain, the drug may, for the time, be discontinued. It is to be prescribed to suit the exigencies of the case. It is unpleasant to have to remark the fact that these drugs, so useful in many cases, will now and again fail in their action; will, in remote cases, occasionally even tend to incite delirium and other objectionable symptoms, such as stomach and kidney derangement, but their great value in well selected cases makes them easily our most reliable agency. It will sometimes be necessary to adminsiter morphine hypodermically to relieve pain, produce sleep and restore comfort, when the family of drugs just mentioned have proved ineffective. The bowels may be moved with compound cathartic pills, and the motions ought to be received in a bedpan, because it hurts the patient so to be forced to get up and down. If he is humanely spared that torture, he will show a grateful appreciation of the kindness. These cases require the services of a gentle attendant. They will not appreciate being told that the pain is slight, and that they must bear it bravely while they are being roughly handled. No solid food should be administered until the tongue becomes clean and evidences of convalescence are marked. Excepting those young subjects who are pale, weak and anæmic, it is well, perhaps, to render the secretions alkaline by giving, say, two drachms of bicarbonate potash set to effervescence by the addition of an ounce of lemon juice, this to be administered every four hours until the urine will turn the red litmus paper a decided blue color. It is claimed for this treatment by Dr. Fuller, that there is not nearly so much danger of the grave heart complications, and that the subsidence of the disease may be looked forward to, on the eleventh day, or thereabout.

In the young, pale and weak subjects, it is probably best to follow the directions of Dr. Reynolds, and give the tincture of the chloride of iron in thirty-drop doses, diluted with a tumblerful of water, to be taken through a quill every four hours. In the fat, florid, whisky drinker, it is probably best to employ the alkaline treatment as outlined by Drs. Fuller and Dickenson.

In the Herculean subjects of the inherited tendency or rheumatic diathesis, it is probably best to rely upon the salicylates, since in such subjects there is but trifling danger of super-inducing any cardiac weakness.

In those cases of hyperpyrexia, where the fever runs suddenly up to 108 or 110 degrees, the cold bath should at once be administered, and superintended by the physician. The patient should be put in the water at a normal temperature, and by the addition of ice or cold water, the temperature should be rapidly lowered to sixty-five degrees; the patient then removed, dried, and replaced in bed. This procedure may save life in cases of hyperpyrexia originating in other diseases. It is certainly to be recommended as the very best and easiest way of reducing destructive high temperatures.

The salicin family of drugs are of no use in the treatment of chronic rheumatism. Alterative tonics are to be depended upon in this form of the disease, together with hot linaments used energetically, hot baths, blisters, massage, and the galvanic current judiciously applied.

Residence in a warm, dry climate is to be recommended, and every means looking to the preservation of the best possible state of health should be exercised. It is a well known fact that the mineral waters of various resorts are of use, both for potable and bathing purposes, very often giving immediate relief and permanent benefit. The requisite character of such water seems to exist in the hot sulphur wells of Bexar county, a suburban resort in the well known dry and warm climate of San Antonio, of this State.

